# Catheter ablation of short-coupled variant of torsade de pointes

**DOI:** 10.1007/s00392-021-01840-z

**Published:** 2021-03-26

**Authors:** Johannes Steinfurt, Babak Nazer, Martin Aguilar, Joshua Moss, Satoshi Higuchi, Markus Zarse, Luca Trolese, Alexander Gressler, Thomas S. Faber, Katja E. Odening, Manfred Zehender, Christoph Bode, Melvin M. Scheinman, Usha B. Tedrow, Harilaos Bogossian

**Affiliations:** 1 Department of Cardiology and Angiology I, University Heart Center Freiburg, Bad Krozingen, Faculty of Medicine, University of Freiburg, Hugstetter Str. 55, 79106 Freiburg, Germany; 2 Knight Cardiovascular Institute, Oregon Health and Science University, Portland, OR USA; 3 Cardiovascular Division, Brigham and Women’s Hospital, Harvard Medical School, Boston, MA USA; 4 Cardiology Division, University of California, San Francisco, San Francisco, CA USA; 5 Department of Cardiology and Angiology, Märkische Kliniken GmbH, Klinikum Luedenscheid, Luedenscheid, Germany; 6 Department of Cardiology, Maastricht University Medical Center (MUMC+) and Cardiovascular Research Institute Maastricht (CARIM), Maastricht, The Netherlands; 7 University Witten/Herdecke, Witten, Germany

**Keywords:** Short-coupled variant of torsade de pointes, Moderator band, Purkinje, 3D-mapping

## Abstract

**Background:**

The short-coupled variant of torsade de pointes (sc-TdP) is a malignant arrhythmia that frequently presents with ventricular fibrillation (VF) electrical storm. Verapamil is considered the first-line therapy of sc-TdP while catheter ablation is not widely adopted. The aim of this study was to determine the origin of sc-TdP and to assess the outcome of catheter ablation using 3D-mapping.

**Methods and results:**

We retrospectively analyzed five patients with sc-TdP who underwent 3D-mapping and ablation of sc-TdP at five different institutions. Four patients initially presented with sudden cardiac arrest, one patient experienced recurrent syncope as the first manifestation. All patients demonstrated a monomorphic premature ventricular contraction (PVC) with late transition left bundle branch block pattern, superior axis, and a coupling interval of less than 300 ms. triggering recurrent TdP and VF. In four patients, the culprit PVC was mapped to the free wall insertion of the moderator band (MB) with a preceding Purkinje potential in two patients. Catheter ablation using 3D-mapping and intracardiac echocardiography eliminated sc-TdP in all patients, with no recurrence at mean 2.7 years (range 6 months to 8 years) of follow-up.

**Conclusion:**

3D-mapping and intracardiac echocardiography demonstrate that sc-TdP predominantly originates from the MB free wall insertion and its Purkinje network. Catheter ablation of the culprit PVC at the MB free wall junction leads to excellent short- and long-term results and should be considered as first-line therapy in recurrent sc-TdP or electrical storm.

**Graphic abstract:**

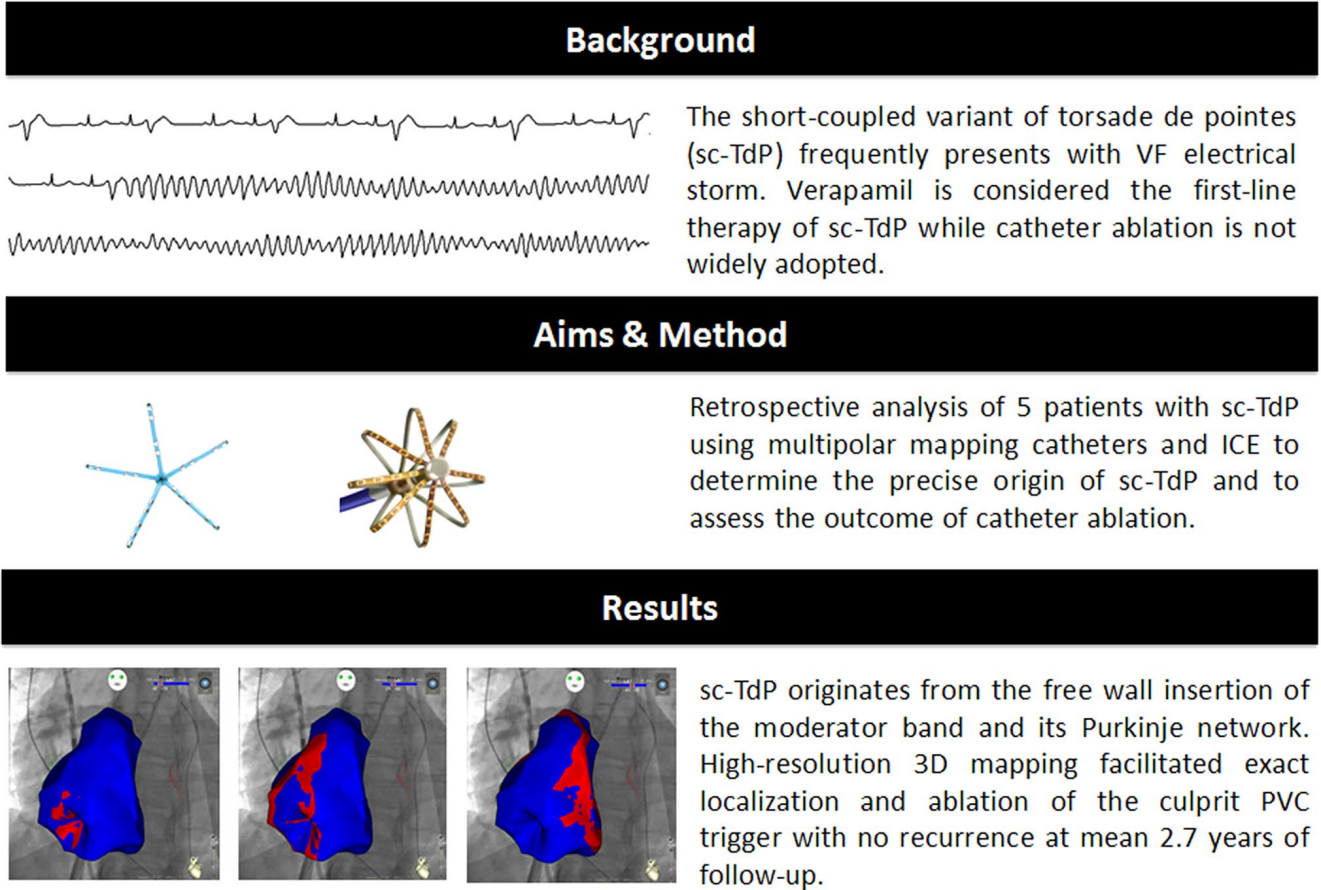

**Supplementary Information:**

The online version contains supplementary material available at 10.1007/s00392-021-01840-z.

## Introduction

The short-coupled variant of torsade de pointes (sc-TdP) was originally described by Leenhardt et al. in 1994 who reported 14 patients with structurally normal hearts, absence of QT interval prolongation, and TdP induced by a premature ventricular contraction (PVC) with late transition left bundle branch block (LBBB) pattern, left superior axis, and a coupling interval of less than 300 ms consistent with a right ventricular (RV) moderator band (MB) origin [1–3]. However, based on the introduction of imaging techniques such as 3D-mapping [4] and intracardiac echocardiography (ICE) [5], sc-TdP and idiopathic ventricular fibrillation (iVF) from the MB have been characterized as different clinical entities which still translates into different therapeutic approaches [1–3]: Verapamil is still considered the first-line therapy in sc-TdP [6] whereas catheter ablation is the treatment of choice for iVF from the MB [2, 3]. Over the past 25 years, numerous reports have highlighted the malignant phenotype of sc-TdP and the insufficient pharmacological treatment (failure of amiodarone, β-blocker, Mg^2+^, lidocaine, procainamide, quinidine, and cilostazol) while catheter ablation is not widely adopted [1, 7–11]. Indeed, one-third of patients with sc-TdP, being treated with either verapamil or β-blockers, die suddenly during a mean follow-up of 7 years [1]. The aim of this study was to assess the precise origin of sc-TdP and to assess the short- and long-term outcomes of catheter ablation of sc-TdP using 3D-mapping and ICE.

## Methods

We retrospectively reviewed the databases of five institutions for sc-TdP treated with 3D-mapping and radiofrequency (RF) catheter ablation. The patients had to have electrocardiographic evidence of sc-TdP, i.e. PVC-induced polymorphic ventricular tachycardia or PVC-induced VF triggered by a monomorphic PVC with late transition LBBB pattern, left superior axis, and a coupling interval of less than 300 ms. Electrophysiological (EP) studies were performed at the discretion of the individual operators. All patients have given written informed consent for publication and ethics approval was not required.

## Results

**Fig. 1:**
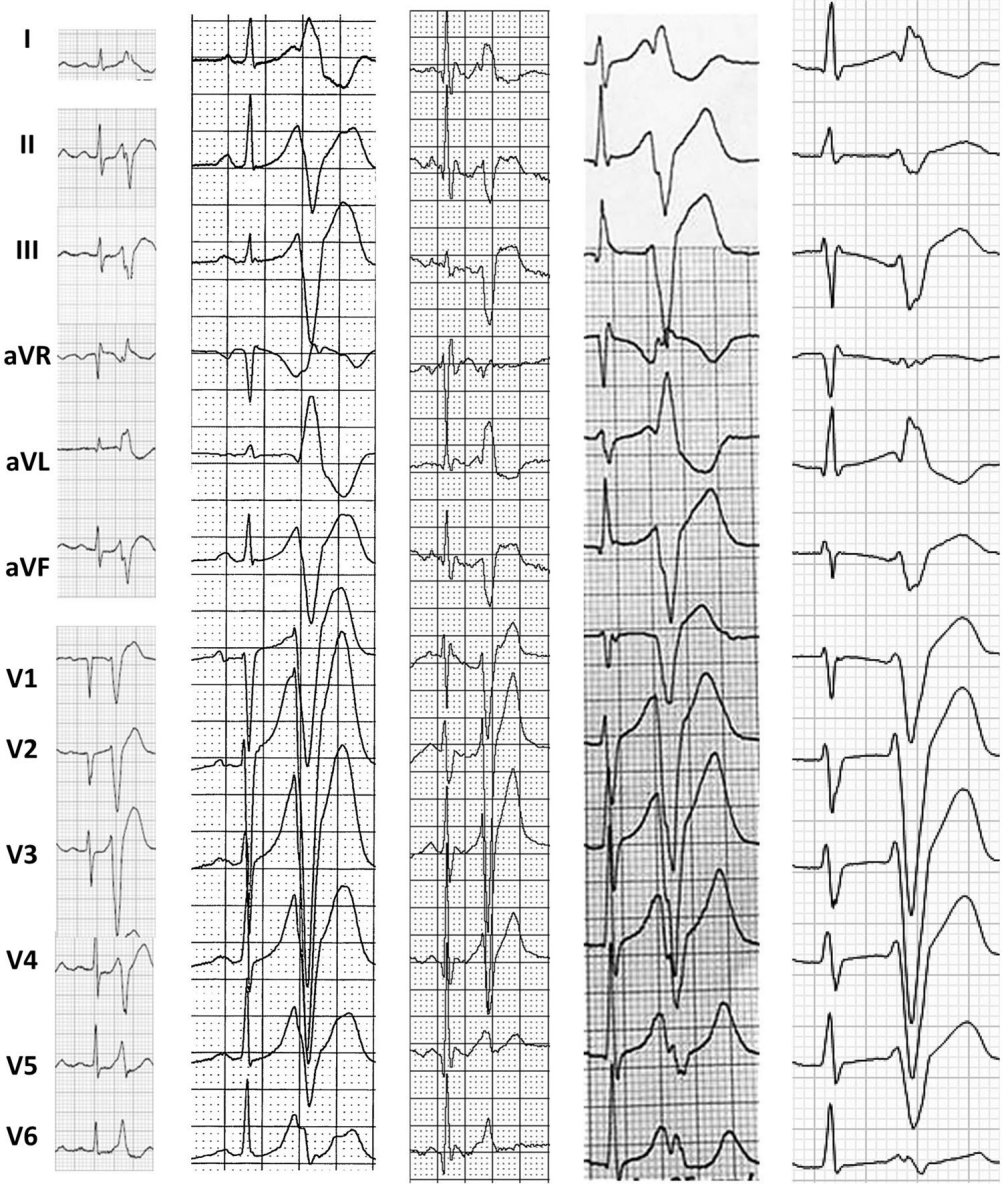
12-lead ECG PVC trigger morphology among five patients with sc-TdP demonstrating a left superior frontal axis (R in I and aVL with *S*_III_ > *S*_II_), late (> V4) precordial transition and a coupling interval < 300 ms. The QRS is relatively narrow (≤ 130 ms.) with fast intrinsicoid deflection (< 60 ms.) indicating an origin within or close to the Purkinje network (paper speed 25 mm/sec)

**Fig. 2:**
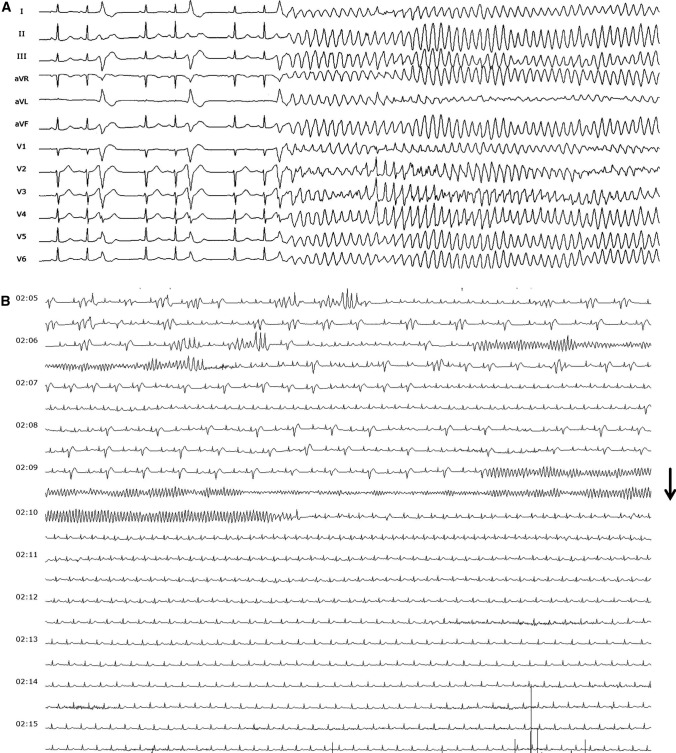
**a**12-lead ECG of sc-TdP in patient #2 who initially experienced arrhythmic syncope. **b** Recurrent sc-TdP in patient #2 which eventually degenerated into VF. Ajmaline bolus (black arrow) was given which led to VF termination and suppression of short-coupled PVCs. Ajmaline infusion (0.8 mg/kg body weight/min) was continued until the next morning when frequent ectopy returned during EP study. Ajmaline can have pro-arrhythmic and fatal consequences when given to patients with Brugada syndrome (BrS) or malignant Purkinje ectopy [27] who may also present with an electrical storm. It is, therefore, essential to recognize the pathognomonic PVC trigger morphology of sc-TdP (late transition, left superior axis with coupling interval < 300 ms). In contrast, VF triggers in BrS show an inferior axis with a longer coupling interval (> 300 ms) [28]

**Fig. 3:**
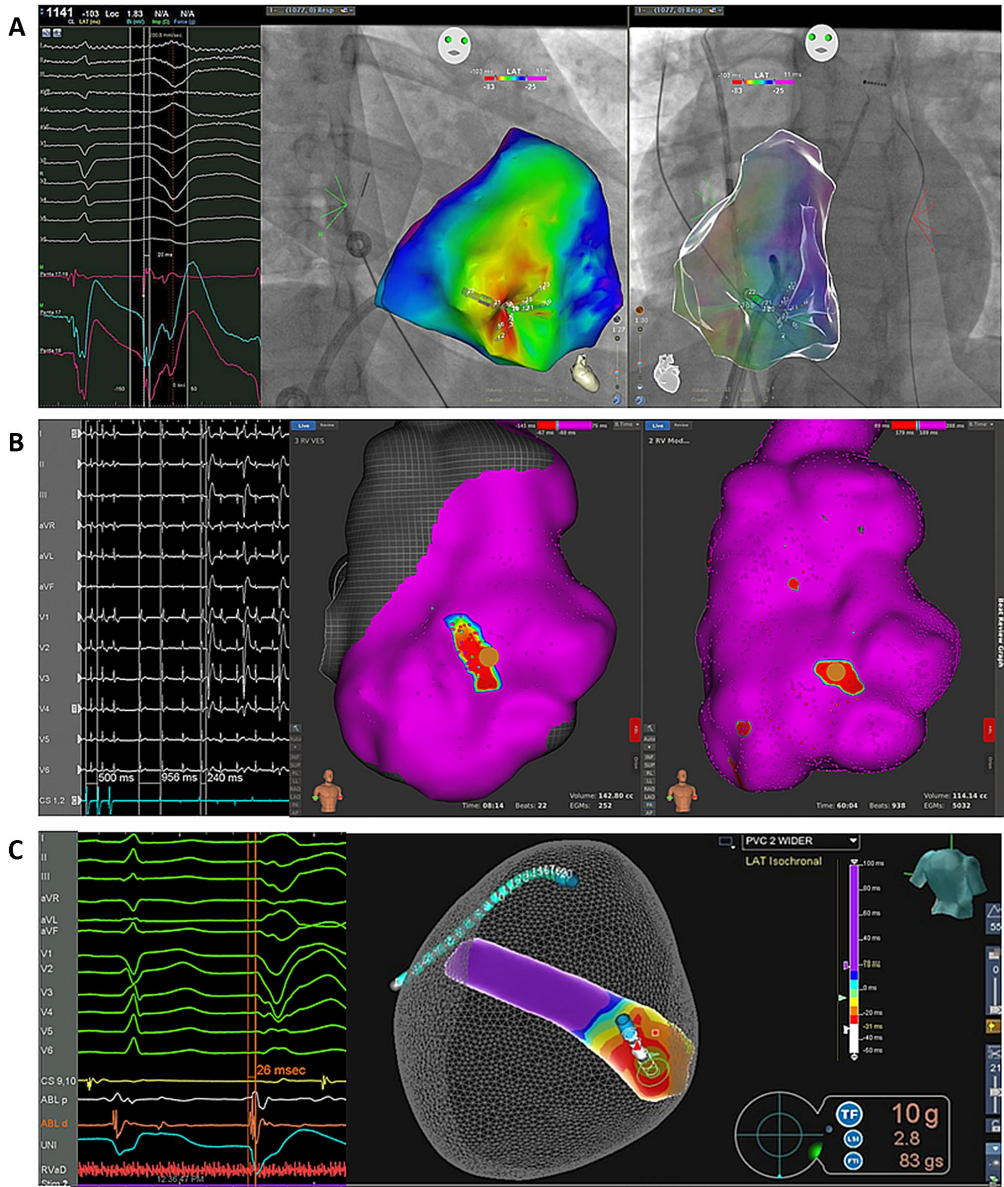
High-resolution 3D activation maps of the culprit PVC in three patients demonstrating earliest activation at the free wall insertion of the MB. **a** The electrode pair (17,18) records a Purkinje potential fused with local activation during sinus rhythm that is separated from local activation and precedes the QRS onset with a unipolar QS pattern by 20 ms. during short-coupled PVC. The course of the MB—spanning from septum to lateral free wall—can be appreciated by the impressions in the electroanatomic shell of the RV. **b** Induction of short-coupled PVCs by atrial overdrive pacing. To locate the MB without ICE the earliest activation of the right ventricular free wall in sinus rhythm (right, brown tag) was mapped matching the earliest activation during short-coupled PVC (brown tag in the left picture), confirming the PVC origin to be at the free wall insertion of the MB. After focal ablation at this site short-coupled PVCs were no longer inducible by atrial pacing. **c** Assuming a RV MB site of origin the MB geometry was created on the electroanatomic mapping system and confirmed by ICE. The earliest activation of the short-coupled PVC was mapped to the free wall insertion of the RV MB, where it was preceded by a prominent Purkinje potential. RF ablation at the site of earliest activation (26 ms pre-QRS) promptly terminated the PVC, and addition ablation was performed at adjacent Purkinje potential sites on the free wall insertion of the MB

We identified five patients (mean age 36.6 ± 13 years) with sc-TdP who underwent 3D-mapping and ablation of sc-TdP between 2011 and 2019 at five different institutions. Four patients initially presented with sudden cardiac arrest, one patient experienced recurrent syncope as the first manifestation of the arrhythmia. Two patients developed electrical storm either during or 2 years after initial admission (Table 1). Structural and ischemic heart disease had been excluded by echocardiography and coronary angiography in all patients. Cardiac MRI was unremarkable in three patients. All patients demonstrated late transition, left superior axis PVCs with extremely short coupling intervals ranging from 280 to 240 ms (Fig. 1) triggering recurrent sc-TdP or VF. Anti–arrhythmic drug therapy was ineffective to control the arrhythmia (Table 1) except for Verapamil and Ajmaline. Ajmaline not only led to the suppression of short-coupled PVCs in patient #2 but also to VF termination (Fig. 2). Three-dimensional electro-anatomical activation mapping of the culprit PVC was performed in all patients. Intracardiac echocardiography (ICE) (AcuNav, Siemens Medical Solutions, USA and Soundstar/Cartosound, Biosense Webster, USA) was available in four patients. In the last three patients high-resolution 3D-mapping, i.e. multipolar mapping catheters with close interelectrode spacing (Pentaray, Biosense Webster, USA; Orion, Boston-Scientific, USA; LiveWire, Abbott; USA) and a steerable sheath (Agilis NxT, Abbott, USA) were used. Ectopy was spontaneous in four and induced by atrial pacing in one patient. In all patients, normal bi- and unipolar voltages without any late or fragmented potentials were demonstrated on endocardial voltage maps. In four patients, the culprit PVC was mapped to the free wall insertion of the moderator band (MB) with a preceding Purkinje potential in two patients (Fig. 3 and Suppl. 1) which were effectively targeted with RF ablation. In one patient without distinct MB the culprit PVC was ablated at a right ventricular (RV) papillary muscle. All patients were implanted with a transvenous (2) or subcutaneous (3) implantable cardioverter-defibrillator (ICD) for secondary prevention. Device interrogations confirmed the elimination of sc-TdP in all patients, with no recurrence at mean 2.7 years (range 6 months to 8 years) of follow-up.

**Table 1:** Study cohort of patients with a short-coupled variant of Torsade de Pointes (sc-TdP), 3D-mapping and catheter ablation at the free wall moderator band (MB) insertion or MB complex

	Patient #1	Patient #2	Patient #3	Patient #4	Patient #5
Age at onset (years), sex	56, f	33, f	22, m	43, m	29, m
History of arrhythmic syncope	No	Yes	No	No	No
Sudden cardiac arrest/death	SCA	No	SCA	SCA	SCA
Short-coupled PVC with LBBB pattern in V1, late precordial transition (≥ V4) and left superior frontal axis	Yes	Yes	Yes	Yes	Yes
Coupling interval [msec]	280	270	240	280	240
Sc-TdP	Yes	Yes	Yes	Yes	Yes
VF	Yes	Yes	Yes	Yes	Yes
Time to electrical storm	2 years	No	No	No	admission
Absence of structural heart disease (normal TTE and coronary angiography or CT)	Yes	Yes	Yes	Yes	Yes
Effective AAD to prevent PVCs and sc-TdP	n.a	Ajmaline	n.a	n.a	Verapamil
Ineffective AAD	n.a	β-blocker	n.a	β -blocker Verapamil	Lidocaine
3D-Mapping	Yes	Yes	Yes	Yes	Yes
Anatomical PVC origin	MB free wall insertion	MB free wall insertion	MB free wall insertion	MB free wall insertion	Inferoseptal right ventricle
Preceding Purkinje potential at MB complex	No	Yes	No	Yes	No
Absence of late potentials	Yes	Yes	Yes	Yes	Yes
Successful catheter ablation of culprit PVC	Yes	Yes	Yes	Yes	Yes
Time free from VF after catheter ablation	8 years without AADs	15 mo without AADs	6 mo without AADs	15 mo without AADs	2.5 years on flecainide

*n.a.* not available

## Discussion

To our knowledge, this is the first study using high-resolution 3D-mapping to specify the free wall insertion of the RV MB as the predominant origin of short-coupled PVCs triggering sc-TdP. As the right bundle branch and its Purkinje network run along with the MB and arborize via the MB at the free wall, it is possible to record myocardial as well as Purkinje potentials in this region as seen in our patients. In one patient ablation was performed at an papillary muscle, i.e. at the MB complex. RV Purkinje VF triggers display an uniform morphology which has first been reported by Haïssaguerre et al. who pioneered mapping and ablation of Purkinje-related idiopathic VF (iVF) [12–14]. In addition, Haïssaguerre et al. provided non-invasive ECG imaging data of RV Purkinje triggers that point to the MB free wall insertion as PVC breakthrough site in these patients [14, 15]. Walton et al. also demonstrated ex vivo that uncoupled myocardial and Purkinje MB cells have different action potential durations facilitating re-entry and short-coupled PVCs in this region [16]. The identical PVC morphology has been found to represent a RV MB origin using ICE in a patient with VF storm [2] and a series of 10 patients with monomorphic VT, VF, and recurrent ICD shocks [3]. According to the recently proposed mechanistic classification of primary arrhythmogenic diseases causing sudden cardiac arrest by Haïssaguerre et al., sc-TdP should be classified as iVF from Purkinje-myocardial sources [17].

Three-dimensional activation mapping is essential for the understanding of complex ventricular arrhythmias and especially in cases of papillary muscle or moderator band sources [18]. However, 3D activation mapping of these endocardial structures may carry certain technical challenges. As seen in one patient, the mapping system may erroneously project internal activation points to the surface geometry (Fig. 3a and Suppl. 1) which can be misleading. Activation mapping of the earliest free wall activation site in sinus rhythm (Fig. 3b) may help to correctly localize the MB free wall junction. The novel point density exclusion (PDX) mapping algorithm (Fig. 3c)—that uses absent 3D mapping points within a ventricular lumen to create an endocavitary geometry—may represent an optimal approach in these cases as it allows to generate a separate internal geometry facilitating precise activation mapping [19]. In addition, as seen in two of our patients, multielectrode mapping catheters with close interelectrode spacing may enhance the detection of Purkinje potentials [20]. To monitor and improve contact and stability during mapping and ablation at the MB free wall junction, the use of ICE integrated into the 3D-mapping system as well as the cryoballoon have been advocated [5, 21]. Finally, pace-mapping, in case of non-inducibility of the clinical PVC, may also yield good results as the Purkinje network is an entirely endocardial structure.

The genesis of sc-TdP is currently incompletely understood. Two recent studies have linked sc-TdP with *RyR-2* mutations leading to either a decreased RYR2 function or increased RYR2 leak which may promote triggered activity and early afterdepolarizations via an increased Ca^2+^ release. The PVC morphologies reported in those two studies are also typical of a MB-free wall origin [22, 23]. The induction of short-coupled PVCs with atrial overdrive pacing and suppression with Verapamil and Ajmaline in our patients also support triggered activity as the underlying mechanism of sc-TdP. The Dutch iVF cohort linked to the *DPP6* risk haplotype shows identical PVC trigger morphologies (Xiao et al., Supp. Online Figure 1), however, based on a different mechanism. In these patients, the *DPP6* mutation results in selectively accelerated Purkinje fiber repolarization which may lead to strong repolarization gradients with adjacent ventricular muscle creating an electrophysiological substrat for re-entry and short-coupled PVCs [24, 25]. The susceptibility of the RV MB free wall junction to generate short-coupled PVCs may also be related to a mechanical stretch of the MB during early diastole `mechanoelectrical feedback’ which may involve stretch-activated ion channels [26]. A link between short-coupled PVCs from the MB and mechanical function is supported by the beneficial effect of mechanical unloading in a single patient with intractable VF due to sc-TdP [8].

## Conclusion

We have demonstrated that sc-TdP predominantly originates from the free wall insertion of the RV MB and its Purkinje network and particularly high-resolution 3D-mapping and ICE may be useful to precisely locate the triggering PVC in this region facilitating successful catheter ablation. Catheter ablation of sc-TdP leads to excellent short- and long-term results and should be considered as first-line therapy in patients presenting with recurrent TdP or electrical storm. Our observations support triggered activity as the underlying mechanism of sc-TdP which should be classified as iVF from Purkinje-myocardial sources.

### Limitations

We are aware of the fact that it may be difficult to draw conclusions from five patients with relatively short follow-up and without a control group of non-ablated patients. However, sc-TdP is an extremely rare entity and our patients show consistent results. Most importantly, our findings may aid in successful catheter ablation of sc-TdP—a potentially life-threatening arrhythmia with a high incidence of electrical storm and intractable VF.

## Supplementary Information

Below is the link to the electronic supplementary material.

S1 Initial septal to lateral and then centrifugal spread of ventricular activation by short-coupled PVC originating from the MB. The lateral impression in the electro-anatomic shell of the RV corresponds to the MB free wall insertion (AVI 2847 KB)

## References

[1] Leenhardt A, Glaser E, Burguera M, Nurnberg M, Maison-Blanche P, Coumel P (1994). Short-coupled variant of torsade de pointes. A new electrocardiographic entity in the spectrum of idiopathic ventricular tachyarrhythmias. Circulation.

[2] Anter E, Buxton AE, Silverstein JR, Josephson ME (2013). Idiopathic ventricular fibrillation originating from the moderator band. J Cardiovasc Electrophysiol.

[3] Sadek MM, Benhayon D, Sureddi R, Chik W, Santangeli P, Supple GE, Hutchinson MD, Bala R, Carballeira L, Zado ES, Patel VV, Callans DJ, Marchlinski FE, Garcia FC (2015). Idiopathic ventricular arrhythmias originating from the moderator band: electrocardiographic characteristics and treatment by catheter ablation. Heart Rhythm.

[4] Ben-Haim SA, Osadchy D, Schuster I, Gepstein L, Hayam G, Josephson ME (1996). Nonfluoroscopic, in vivo navigation and mapping technology. Nat Med.

[5] Enriquez A, Saenz LC, Rosso R, Silvestry FE, Callans D, Marchlinski FE, Garcia F (2018). Use of intracardiac echocardiography in interventional cardiology: working with the anatomy rather than fighting it. Circulation.

[6] Chokr MO, Darrieux FC, Hardy CA, Hachul DT, Britto AV, Melo SL, Pisani C, Sosa EA, Martinelli Filho M, Scanavacca MI (2014). Short-coupled variant of "torsades de pointes" and polymorphic ventricular tachycardia. Arq Bras Cardiol.

[7] Shiga T, Shoda M, Matsuda N, Fuda Y, Hagiwara N, Ohnishi S, Watanabe A, Kasanuki H (2001). Electrophysiological characteristic of a patient exhibiting the short-coupled variant of torsade de pointes. J Electrocardiol.

[8] Durand-Dubief A, Burri H, Chevalier P, Touboul P (2003). Short-coupled variant of torsades de pointes with intractable ventricular fibrillation: lifesaving effect of cardiopulmonary bypass. J Cardiovasc Electrophysiol.

[9] Bogaard K, van der Steen MS, Tan HL, Tukkie R (2008). Short-coupled variant of torsade de pointes. Neth Heart J.

[10] Chiladakis JA, Spiroulias G, Koutsogiannis N, Zagli F, Alexopoulos D (2008). Short-coupled variant of Torsade de Pointes as a cause of electrical storm and aborted sudden cardiac death: insights into mechanism and treatment. Hell J Cardiol.

[11] Burrows K, Fox J, Biblo LA, Roth JA (2013). Pregnancy and short-coupled torsades de pointes. Pacing Clin Electrophysiol.

[12] Haissaguerre M, Shoda M, Jais P, Nogami A, Shah DC, Kautzner J, Arentz T, Kalushe D, Lamaison D, Griffith M, Cruz F, de Paola A, Gaita F, Hocini M, Garrigue S, Macle L, Weerasooriya R, Clementy J (2002). Mapping and ablation of idiopathic ventricular fibrillation. Circulation.

[13] Knecht S, Sacher F, Wright M, Hocini M, Nogami A, Arentz T, Petit B, Franck R, De Chillou C, Lamaison D, Farre J, Lavergne T, Verbeet T, Nault I, Matsuo S, Leroux L, Weerasooriya R, Cauchemez B, Lellouche N, Derval N, Narayan SM, Jais P, Clementy J, Haissaguerre M (2009). Long-term follow-up of idiopathic ventricular fibrillation ablation: a multicenter study. J Am Coll Cardiol.

[14] Haïssaguerre M, Vigmond E, Stuyvers B, Hocini M, Bernus O (2016). Ventricular arrhythmias and the His-Purkinje system. Nat Rev Cardiol.

[15] Cheniti G, Vlachos K, Meo M, Puyo S, Thompson N, Denis A, Duchateau J, Takigawa M, Martin C, Frontera A, Kitamura T, Lam A, Bourier F, Klotz N, Derval N, Sacher F, Jais P, Dubois R, Hocini M, Haissaguerre M (2018). Mapping and ablation of idiopathic ventricular fibrillation. Front Cardiovasc Med.

[16] Walton RD, Pashaei A, Martinez ME, Constantin M, Duchateau J, Bear L, Cros C, Pascarel-Auclerc C, Guo Y, Benoist D, Dubes V, Faye NR, Chaigne S, Dupuis S, Détaille D, Pourtau L, Pasdois P, Brette F, Rogier J, Labrousse L, Hocini M, Vigmond EJ, Haïssaguerre M, Bernus O (2018). Compartmentalized structure of the moderator band provides a unique substrate for macroreentrant ventricular tachycardia. Circ Arrhythm Electrophysiol.

[17] Haïssaguerre M, Nademanee K, Hocini M, Cheniti G, Duchateau J, Frontera A, Sacher F, Derval N, Denis A, Pambrun T, Dubois R, Jaïs P, Benoist D, Walton RD, Nogami A, Coronel R, Potse M, Bernus O (2019). Depolarization versus repolarization abnormality underlying inferolateral J-wave syndromes: new concepts in sudden cardiac death with apparently normal hearts. Heart Rhythm.

[18] Kim YH, Chen SA, Ernst S, Guzman CE, Han S, Kalarus Z, Labadet C, Lin YJ, Lo LW, Nogami A, Saad EB, Sapp J, Sticherling C, Tilz R, Tung R, Kim YG, Stiles MK (2020). 2019 APHRS expert consensus statement on three-dimensional mapping systems for tachycardia developed in collaboration with HRS, EHRA, and LAHRS. J Arrhythm.

[19] Miller JD, Dewland TA, Henrikson CA, Reiss J, Patel A, Nazer B (2020). Point density exclusion electroanatomic mapping for ventricular arrhythmias arising from endocavitary structures. Heart Rhythm O2.

[20] Ho RT, Frisch DR, Greenspon AJ (2017). Idiopathic ventricular fibrillation ablation facilitated by PENTARAY mapping of the moderator band. JACC Clin Electrophysiol.

[21] Chinitz JS, Sedaghat D, Harding M, Darge A, Epstein LM, John R (2019). Adjuvant use of a cryoballoon to facilitate ablation of premature ventricular contraction-triggered ventricular fibrillation originating from the moderator band. HeartRhythm Case Rep.

[22] Cheung JW, Meli AC, Xie W, Mittal S, Reiken S, Wronska A, Xu L, Steinberg JS, Markowitz SM, Iwai S, Lacampagne A, Lerman BB, Marks AR (2015). Short-coupled polymorphic ventricular tachycardia at rest linked to a novel ryanodine receptor (RyR2) mutation: leaky RyR2 channels under non-stress conditions. Int J Cardiol.

[23] Fujii Y, Itoh H, Ohno S, Murayama T, Kurebayashi N, Aoki H, Blancard M, Nakagawa Y, Yamamoto S, Matsui Y, Ichikawa M, Sonoda K, Ozawa T, Ohkubo K, Watanabe I, Guicheney P, Horie M (2017). A type 2 ryanodine receptor variant associated with reduced Ca(2+) release and short-coupled torsades de pointes ventricular arrhythmia. Heart Rhythm.

[24] Xiao L, Koopmann TT, Ordog B, Postema PG, Verkerk AO, Iyer V, Sampson KJ, Boink GJ, Mamarbachi MA, Varro A, Jordaens L, Res J, Kass RS, Wilde AA, Bezzina CR, Nattel S (2013). Unique cardiac Purkinje fiber transient outward current beta-subunit composition: a potential molecular link to idiopathic ventricular fibrillation. Circ Res.

[25] Wilde AAM, Garan H, Boyden PA (2019). Role of the Purkinje system in heritable arrhythmias. Heart Rhythm.

[26] Quinn TA, Jin H, Lee P, Kohl P (2017). Mechanically induced ectopy via stretch-activated cation-nonselective channels is caused by local tissue deformation and results in ventricular fibrillation if triggered on the repolarization wave edge (commotio cordis). Circ Arrhythm Electrophysiol.

[27] William Escande, Jean Baptiste Gourraud, Josselin Duchateau, Jose Luis. Merino, Xavier Waintraub, Ghassen Cheniti, Estelle Gandjbakhch, Christophe I. Leclercq, Thomas Lavergne, Anne-Marie Chac, Jean-Sylvain Hermida, Philippe Maury, Anna Lam, Masateru Takigawa, Clementine Andre, Thomas Pambrun, Arnaud Denis, Nicolas Derval, Koonlawee Nademanee, Meleze Hocini, Pierre Jais, Frederic Sacher, Olivier Bernus, Vincent Probst, Michel Haissaguerre (2019) Heart Rhythm 16(5):S330–S425. 10.1016/j.hrthm.2019.04.017

[28] Maury P, Hocini M, Haissaguerre M (2005). Electrical storms in Brugada syndrome: review of pharmacologic and ablative therapeutic options. Indian Pacing Electrophysiol J.

